# Supplementary material for: Cerebellar Transcriptomic Analysis in a Chronic plus Binge Mouse Model of Alcohol Use Disorder Demonstrates Ethanol-Induced Neuroinflammation and Altered Glial Gene Expression
Source: Cells. 2023 Feb 25;12(5):745. doi: 10.3390/cells12050745 (PMC10000476; doi:10.3390/cells12050745)
Supplement: Supplementary file 1 [file cells-12-00745-s001.zip › cells-2193483 supplementary materials.pdf]

Table S1A. Microglia associated genes [identified in (29-33)].

0610040J01RIK	AFTPH	ARHGDIB	C1QB	CD200R1	CLEC5A	CXCL10	DOK3	EVI2B	FTL1	GPR160	HK2
1700017B05RIK	AGO4	ARHGEF6	C1QC	CD209F	CLIC1	CXCL16	DRAM1	F11R	FYB	GPR183	HK3
1810011H11RIK	AHR	ARL4C	C3AR1	CD209G	CLIP1	CXCL2	DSE	F13A1	G530011O06RIK	GPR34	HMGA2-PS1
4632428N05RIK	AI607873	ARPC1B	C5AR1	CD2AP	CLTA	CYBA	DSN1	FAM102B	GAL3ST4	GPR65	HMHA1
4933406I18RIK	AI662270	ARPC2	CAMK1	CD300A	CLTC	CYBB	DTX2	FAM105A	GALNT12	GPR84	HMHA1
5430427O19RIK	AIF1	ARRB2	CAPN3	CD33	CMAH	CYFIP1	DTX4	FAM111A	GAS6	GPX1	HMOX1
6230400D17RIK	AIM2	ARRB2	CAPZA2	CD34	CMTM6	CYSLTR1	DUSP1	FAM134B	GCNT1	GPX3	HPGD
9830001H06RIK	AKAP13	ARSB	CASP1	CD36	COL27A1	CYTH4	DUSP6	FAM46A	GDF15	GRAP	HPGDS
A630001G21RIK	AKR1B10	ASAH1	CASP4	CD37	COMMD8	CYTH4	E230029C05RIK	FAM46C	GDI2	GRN	HPS3
AB124611	ALOX5	ASAP1	CASP8	CD48	COPS4	D15ERTD621E	EBI3	FAM49B	GDPD3	GTF2H2	HPS4
ABCA1	ALOX5AP	ASPH	CASS4	CD53	CORO1A	D17H6S56E-5	EDEM1	FAU	GEM	GUSB	HSPA1A
ABCA9	AMDHD2	ATF3	CBFA2T3	CD68	COTL1	D830030K20RIK	EDEM2	FBXW4	GINS2	H2-AA	HSPA1B
ABCB1B	ANG	ATF4	CBR2	CD82	CRADD	DAB2	EFHD2	FCER1G	GIT2	H2-D1	HSPA5
ABCC3	ANGPTL7	AXL	CCDC50	CD83	CREG1	DAGLB	EGR1	FCGR1	GM10336	H2-DMB1	HSPB1
ABCD2	ANXA3	B2M	CCDC86	CD84	CRYL1	DAZAP2	EGR2	FCGR2B	GM13139	H2-K1	HVCN1
ABHD12	AP1B1	BAZ1A	CCDC88B	CD86	CSF1R	DCLRE1C	EHBP1L1	FCGR3	GM13476	H2-Q10	ICAM1
ABHD15	APAF1	BC035044	CCDC9	CDKN1A	CSF2RB	DDX26B	EHD1	FCNA	GM14548	H2-Q2	IER2
ABI3	APBB1IP	BCL2A1B	CCL12	CEBPA	CSF3R	DDX3X	EHD4	FCRLS	GM20605	H2-T23	IER3
ABL1	APH1C	BCL2A1C	CCL2	CEBPB	CSK	DDX5	EIF5	FERMT3	GM2382	H3F3B	IER5
ACP2	APOBEC1	BHLHE41	CCL24	CENPF	CSRNP1	DENND1B	ELF1	FEZ2	GM3002	H60C	IFFO1
ACP5	APOBEC3	BID	CCL3	CEP110	CST3	DENND1C	ELK3	FGD2	GM5086	HAVCR2	IFI202B
ACVR1	APOC1	BIN1	CCL4	CERK	CTSA	DENND4A	ELMO1	FMNL1	GM6377	HCK	IFI203
ADA	ARAP3	BIN2	CCL6	CFH	CTSB	DHDH	ELMSAN1	FMNL3	GM8615	HCLS1	IFI204
ADAM10	ARHGAP11A	BLNK	CCL7	CFHR2	CTSC	DHRS3	EML3	FMO5	GMFG	HERC2	IFI27L2A
ADAM15	ARHGAP17	BLVRA	CCL9	CFP	CTSD	DIAP2	EMR1	FNDC3A	GNG10	HERPUD1	IFNAR2
ADAM17	ARHGAP19	BLVRB	CCNL1	CH25H	CTSH	DNAJB1	ENG	FNIP2	GNGT2	HEXA	IFNGR1
ADAM33	ARHGAP25	BMP2	CCR1	CITED2	CTSL	DNAJB4	ENTPD1	FOLR2	GNPDA1	HEXB	IFNGR2
ADAMTS1	ARHGAP27	BRD2	CCR5	CLCN5	CTSS	DNASE2A	EPB41L2	FOS	GNS	HFE	IFRD1
ADAP2	ARHGAP30	BTG1	CCRL2	CLEC10A	CTSZ	DNM2	EPSTI1	FOSB	GOLM1	HHEX	IGSF6
ADCY7	ARHGAP39	BTG2	CD14	CLEC4A2	CTTNBP2NL	DOCK10	ERP29	FRMD4A	GP49A	HIST1H2AE	IKZF1
ADRB2	ARHGAP4	BTK	CD163	CLEC4A3	CX3CR1	DOCK2	ETHE1	FRRS1	GPNMB	HIST1H3I	IL10RA
AF251705	ARHGAP5	C1QA	CD164	CLEC4N	CXCL1	DOCK8	EVI2A	FSCN1	GPR155	HIST2H2BB	IL16

IL17RA	KLF6	LY96	MILR1	NCKAP1L	P2RX7	PLOD3	PYCARD	RNF19B	SH2B2	SNX2	TBXAS1	TNFRSF21	VCAN
IL18BP	KLHL6	LYL1	MIR1903	NCKAP5L	P2RY12	PLXDC2	PYROXD2	RNF19B	SH2B3	SNX5	TCIRG1	TNFSF9	WDFY4
IL1A	KRCC1	LYN	MIR692-1	NCOA3	P2RY13	PMEPA1	RAB11FIP5	RNU12	SH3BP1	SOCS3	TCN2	TOR1AIP1	WFDC17
IL1B	LAIR1	LYVE1	MKI67	NDST2	P2RY6	PMF1	RAB32	RP2H	SH3GLB1	SP110	TFEC	TPM3	WSB1
IL4RA	LAPTM5	LYZ1	MKNK1	NEK6	PACSIN2	PMP22	RAB39	RPL35A	SHCBP1	SPARC	TGFBI	TPP1	WWP1
IL6RA	LAT2	LYZ2	MLEC	NEURL3	PAG1	PNP	RAB3IL1	RPLP1	SIGLECH	SPI1	TGFBR1	TREM2	XBP1
IL6ST	LCP1	MAF	MLXIPL	NFAM1	PALD1	PNRC1	RAB43	RPS6KA1	SIPA1	SPIDR	TGFBR2	TRIB1	XLR
IL7R	LCP2	MAFB	MLXIPL	NFATC2	PAOX	POLA1	RAB8B	RRBP1	SIRPA	SPP1	TGIF1	TRIM21	YWHAH
INPP5D	LEPREL1	MAFF	MNDA	NFE2L2	PARVG	POU2F2	RAC2	RRP1B	SKAP2	SPTY2D1	TIFAB	TRIM47	ZC3H12C
IRAK4	LGALS3BP	MAML1	MOCOS	NFKB1	PCF11	PPCDC	RAD51AP1	RTN4RL1	SKI	SRGAP2	TIMP2	TSC22D3	ZCCHC2
IRF1	LGALS9	MAN1C1	MORC3	NFKBIA	PDE3B	PPFIA4	RAP1B	SALL1	SKIL	SRGAP2	TLN1	TSLP	ZFAND5
IRF2BPL	LGMN	MAN2B1	MPEG1	NFKBID	PDGFB	PPP1R15A	RAP1GDS1	SALL3	SLA	SRGN	TLN2	TSPAN14	ZFHX3
IRF5	LHFPL2	MAP2K1	MPP1	NFKBIZ	PF4	PPP1R18	RASGRP3	SAMSN1	SLAMF9	SRSF5	TLR13	TSPO	ZFP110
IRF7	LILRA5	MAP3K14	MRC1	NFXL1	PHF15	PRCP	RBPJ	SASH3	SLC11A1	SSH2	TLR2	TUBA1C	ZFP36
IRF8	LILRB4	MAP3K8	MS4A6B	NHLRC3	PHYHD1	PRKAB1	RCAN1	SAT1	SLC15A3	ST3GAL5	TLR3	TUBB6	ZFP36L1
ISYNA1	LIMD2	MAPK14	MS4A6C	NINJ1	PICALM	PRKCD	RCBTB2	SBNO2	SLC16A6	ST3GAL6	TLR7	TUBGCP5	ZFP36L2
ITGAM	LIMS1	MAPKAPK2	MS4A6D	NLRP3	PIK3CD	PROS1	REL	SCAMP2	SLC25A5	STAB1	TMCO4	TUFT1	ZFP467
ITGB2	LMO2	MARCKS	MS4A7	NMT1	PIK3CG	PRPSAP2	RENBP	SCEL	SLC29A3	STAB1	TMEM106A	TXNIP	ZFP568
ITGB5	LPAR6	MASP2	MSN	NNT	PIK3R5	PSAP	RGL2	SCOC	SLC2A5	STAG3	TMEM119	TYMS	ZFP710
ITM2B	LPCAT2	MB21D1	MSR1	NPL	PIP4K2A	PSMB8	RGS1	SCPEP1	SLC37A2	STARD3	TMEM173	TYROBP	ZFP90
ITSN1	LPCAT3	MBNL1	MTDH	NRP1	PIRA4	PTAFR	RGS10	SELPLG	SLC38A6	STK10	TMEM55B	UAP1L1	ZSWIM6
IVNS1ABP	LPIN2	MCFD2	MTR	NRROS	PIRB	PTBP1	RGS19	SEMA4D	SLC7A7	STK3	TMEM63A	UBASH3B	ZYX
JAK1	LPL	MCL1	MTUS1	NTPCR	PISD-PS3	PTBP3	RGS2	SEPP1	SLC7A8	SUSD1	TMEM64	UBC	
JMJD1C	LPXN	MEF2A	MYD88	NUAK2	PKN1	PTGS1	RGS3	SERINC3	SLC9A9	SUSD3	TMEM8	UBE2J1	
JUN	LRBA	MEF2C	MYLIP	NUMB	PLA2G15	PTP4A3	RHOB	SERPINB8	SLCO2B1	SYK	TMEM86A	UCP2	
JUNB	LRMP	MERTK	MYO1F	OAS2	PLA2G4A	PTPLAD2	RHOG	SERPINF1	SLFN2	SYNGR2	TMSB4X	UGT1A2	
JUND	LRP6	METTL20	MYO1F	OGFRL1	PLAGL2	PTPN1	RHOH	SFPI1	SLMAP	TAB2	TNF	UGT1A7C	
KCTD12	LRRC3	MFAP3	NAGPA	OLFML3	PLAU	PTPN18	RILPL2	SFT2D1	SMAD3	TAGAP	TNFAIP3	UNC93B1	
KDM2B	LST1	MFSD1	NAIP5	ORAI1	PLCG2	PTPN6	RIN2	SFT2D2	SMAD7	TAGAP1	TNFAIP8	USP2	
KHK	LTC4S	MGAT1	NCF1	OSBPL11	PLCL2	PTPRC	RNASE4	SGK1	SMAP2	TANC2	TNFAIP8L2	VAMP3	
KLF2	LY6E	MGAT4A	NCF2	OTUD1	PLD4	PTPRJ	RNASEL	SGPL1	SNORD17	TAPBP	TNFRSF1A	VASP	
KLF4	LY86	MGL2	NCF4	P2RX4	PLEK	PXN	RNF130	SGSH	SNX18	TBC1D16	TNFRSF1B	VAV1	

Table S1B. Microglia homeostatic genes [identified in (35 and 37)].

Homeostatic		
ABCC3	IL10RB	SCAMP5
ABI3	IL21R	SERINC3
ADGRG1	INPP4B	SIGLECH
ARHGAP5	ITGA6	SLC2A5
ATP8A2	JUN	SLCO2B1
BASP1	KCTD12	SMAD3
BIN1	LAIR1	ST3GAL6
CCR5	LRRC3	TGFB1
CD33	LTC4S	TGFBR1
CKB	MAFB	TGFBR2
CMKLR1	MEF2A	TJP1
CRYBB1	MERTK	TMEM119
CSF1R	NFKB1	USP2
CST3	NR3C1	X99384
CTTNBP2NL	NRIP1	
CX3CR1	NUAK1	
CXXC5	OLFML3	
EGR1	OPHN1	
F11R	P2RY12	
FCRLS	P2RY13	
FGD2	PDE3B	
FSCN1	PLA2G15	
GLUL	PLXDC2	
GOLM1	PMEPA1	
GPR34	PU.1<br>ORSPI1	
GPR56	RAB3IL1	
GTF2H2	RAP1GDS1	
HEXB	RGMB	
HPGDS	RHOB	
IL10RA	SALL1	

Table S1C. Common microglia genes affected during disease states [identified in (35-37)].

Neurodegenerative		
ALCAM	FABP5	TIMP2
ANK	FER1L3	TLR2
APOE	FTH1	TREM2
ARG1	GAS7	TYROBP
AXL	GNAS	VEGFA
B2M	GPNMB	
BHLHE40	GPX3	
CADM1	GRN	
CCL2	GUSB	
CCL6	H2-D1	
CCRL2	HIF1A	
CD52	IFI202B	
CD63	IGF1	
CD68	IL1B	
CD9	ITGAX	
CFP	LAG3	
CHI3L3	LGALS3	
CLEC7A	LILRB4	
CSF1	LIRB4	
CST7	LPL	
CSTB	LYZ2	
CTSA	MIF	
CTSB	MIR155	
CTSD	MSR1	
CTSL	NOS2	
CTSZ	SERINC3	
CXCL10	SERPINE2	
CXCL16	SIGLEC1	
CXCR4	SPP1	
CYBB	TFEC	

Table S2. Categorized astrocyte associated genes [identified in (76)].

**Acute Injury**							**Pan-Injury**			**Chronic Neurodegenerative Diseases**	
ADIPOR1	REXO2	GABARAP	PSMD9	ABR	VCL	TBL1X	GFAP	GAP43	STAT2	CAPN3	SENP8
ATP11A	RNF4	GABPA	RALB	ACVR2B	VEGFB	THSD7A	CSF1	LAPTM5	ZNFX1	LAMA4	SOX2
CD63	SGPL1	GALNT2	RER1	ANGEL2	WASF3	TMEM107	IFIT3	LGALS3	IDI1	COBL	STT3B
CTSA	SLC41A2	GBA	RNF141	ASF1A	ARNTL	TTC39B	AHNAK	PSME1	INSIG1	ESYT1	TMEM229A
GFPT2	TFG	GSS	SCAMP4	BAZ2A	BRCC3	YIPF4	CD44	ARHGDIB	RAB30	KLC1	TMEM41B
HAT1	TMEM65	HINT1	SGCB	CLASP2	CDCC113	ZBTB24	CEBPD	ATF3	TIMP3	PEX5L	TOM1L1
MTMR9	USP14	IARS	SLC44A3	CPSF6	CDK5		GPNMB	CDKN1A	ACSS2	TSC22D1	WDR17
NLN	XRCC6	IL33	SNAP23	CYR61	CNBP		STAT3	CPNE8	FUT9	APP	ACOT6
PUS7L	YWHAZ	IMP4	SNX10	FUT8	DMD		TIMP1	CTSH	FZD2	CYR61	DCK
ANKRD49	ARL4C	INTS7	SNX33	GNAL	EP300		VIM	FAS	HMGCS1	GATAD2B	DR1
ANXA5	ARRDC4	IPO5	SSR3	GPRASP1	IRF2		C3	IFI44	PPARGC1A	NR4A3	INHBB
CCNG1	AXL	KARS	TMEM176B	KALRN	KLHL3		CH25H	IFIH1	PTPRD	RUSC1	NFYC
CCT4	BCL10	LAP3	TMEM206	LRP1B	MAPK9		CTSS	IRF9	TTPA	THBS4	PATZ1
CDC42	CALU	LRRC41	TOR1AIP2	LRRK2	MSRB2		CYB5R3	LGALS1	BBOX1	FAM69C	SOD2
CDC42SE1	CARS	LRRC58	TRIM21	MECP2	NCDN		GPX1	LGALS3BP	ELOVL2	S1PR1	TMEM185B
DEDD	CCT2	LSM4	UBE2F	MYCBP2	NIPBL		HSPB1	MOBP	NSDHL	TRIB2	
ESD	CDK4	MCFD2	WDSUB1	NIT1	NR2F2		IQGAP1	NFASC	AHCYL1	AGL	
FARSB	CFL1	MFSD1	YKT6	NR2C2	NUMB		MCL1	RPS4X	APLN	ARSK	
IBTK	CHMP7	MYCBP	CBX7	OTUD5	PIP5K1B		OSMR	SH3BP2	ARHGEF9	CD164	
LAMP2	CPD	NEDD1	RORA	PCM1	PNPLA8		PARP14	STK3	CDH10	GPR37L1	
LGALS8	CYBRD1	NIPSNAP3A	CRIM1	PDGFRB	PSD2		PARP3	UCP2	GLRB	HEATR3	
LRRC59	DDOST	NOL11	HIVEP1	PPM1B	PTPRG		RHOC	DDX58	SQLE	IGDCC4	
LSG1	DNAJC14	OTUD6B	IGSF1	PRDM2	RBM26		RHOJ	FCER1G	TLCD1	KLHL32	
LYN	DNTTIP2	PEX12	NCOR1	PRELP	RCAN2		SERPING1	GOLM1		NAA30	
NFE2L2	DPH2	PIH1D1	NTRK3	PYGO1	RNF44		STAT1	HAVCR2		PRRX1	
OTUD7B	EEF1E1	POLD4	PSPC1	SAP30L	RPA3		A2M	JUNB			
PLIN2	EIF2B2	PPR1R1A	PTPRN2	SRPK2	SLC25A23		ACTB	NFIL3			
PRRC1	EIF2B3	PPP4C	STAG2	SYNE1	SLC40A1		CEBPB	PARP9			
PSMD2	ENOX2	PPP6C	UBR1	THOC2	SMARCA2		DST	PLCE1			
RELA	FYN	PSMD11	XPA	TSC22D1	SSBP3		FLOT1	PLEK			

Table S3. Categorized oligodendrocyte associated genes [identified in (29, 30, and 38)].

**Oligodendrocyte Progenitor Cell Associated Genes**										
0610040J01RIK	ASIC1	CMTM4	FAM126B	GRIA4	LRRN1	NLGN1	PNMAL2	SCRG1	STRN4	UNC5C
1700025G04RIK	ASRGL1	CNTN1	FAM155A	GRIN3A	LRRTM1	NLGN2	POLG	SDC3	SULF2	USP24
1810041L15RIK	ASTN1	COBL	FAM181B	GRM5	LRRTM3	NMNAT2	POU3F1	SEMA3D	SUSD5	VCAN
2310044G17RIK	ATCAY	COL9A3	FAM19A5	HES5	LRRTM4	NOVA1	PPAPDC1A	SEMA5A	SVIL	VLDLR
2900011O08RIK	B3GAT1	CPQ	FAM212B	HIP1	LUZP2	NRCAM	PPFIBP1	SEMA5B	TAGLN2	WSCD1
3110035E14RIK	BAI1	CSMD3	FAM3C	HIP1R	MAGEE1	NRXN1	PRKCB	SEMA6A	TCEAL1	ZCCHC24
3632451O06RIK	BC068157	CSPG4	FAM5C	HSD17B12	MAGEH1	NRXN2	PRKCQ	SERINC5	TCEAL3	ZFP462
4833424O15RIK	BCAN	CSPG5	FAT1	IFI27L1	MANEAL	NTRK3	PTDSS1	SERPINA3N	TDRD7	ZFP488
9530091C08RIK	BMP7	CSTF2	FBXO7	IFIT2	MAP3K1	OLIG1	PTN	SERPINE2	TM7SF3	ZFP945
A730017C20RIK	C1QL1	CTHRC1	FCHSD2	IGFBP7	MATN4	OLIG2	PTPRE	SH3BP4	TMCO3	
A930009A15RIK	C1QL2	CTNND2	FIP1L1	IGSF21	MDGA2	OMG	PTPRG	SH3D19	TMEM100	
ABCG1	C77370	CXADR	FRY	ITGAV	MEG3	ORAI1	PTPRK	SHC4	TMEM132B	
ABHD17B	CACNB4	CYP2J6	G0S2	ITPR2	MEGF11	OSTF1	PTPRN	SLC1A1	TMEM163	
ABI2	CADM2	CYP2J9	GABRB2	JAM2	MFSD2A	P2RX7	PTPRZ1	SLC22A15	TMEM167	
ABR	CADM4	DBC1	GABRB3	JAM3	MIDN	PAK4	PXDC1	SLC22A17	TMEM176A	
ABTB2	CALCRL	DCAF12L1	GAD1	KANK1	MIF4GD	PAPSS1	QPCT	SLC22A23	TMEM176B	
ACAP3	CALY	DDAH1	GALNT13	KBTBD11	MMP15	PCDH10	RAB31	SLC35F1	TMEM179	
ADAM9	CAR11	DISP2	GALNT16	KCND2	MMP16	PCDH11X	RABGAP1L	SLC38A3	TMEM246	
ADCYAP1R1	CAR8	DLGAP1	GALNT2	KCND3	MMP2	PCDH15	RAMP1	SLC7A3	TMEM255A	
ADORA1	CASK	DNER	GAP43	KCNIP3	MPPED2	PCDH17	RAP1GAP	SLITRK1	TMEM255B	
AFAP1L2	CASKIN1	DNM3	GGH	KCNK2	MPZL1	PCDH20	RAPGEF3	SLITRK2	TMEM59L	
AGPAT5	CASKIN2	DOCK9	GJC3	KCNK3	MRPS7	PCDH7	RAPH1	SMOC1	TMEM63B	
AI414108	CAV1	DPP6	GLTP	KLHL13	MTSS1L	PCDH9	RASA3	SMOX	TNR	
AI854517	CAV2	DSCAM	GM13889	KLHL5	MYO10	PCDHB9	RCN1	SNAP91	TOX3	
ALCAM	CCDC114	DSCAML1	GM2A	LAPTM4B	MYO5A	PCMTD2	REV3L	SNX1	TPM1	
AMPD3	CCDC88A	DYNLT3	GNB4	LBH	MYT1	PCSK1N	RGCC	SNX22	TRAF4	
AMZ1	CCND1	EDNRB	GNG3	LGALSL	NACC2	PDE2A	RHOC	SORCS1	TRIB2	
ANKRD11	CD9	EHD3	GNPTG	LHFPL3	NAP1L5	PDGFRA	RLBP1	SORCS3	TRIL	
ANKS1B	CDH11	EMID1	GPC5	LIMD1	NAV1	PGP	RNF144A	SOX10	TRIO	
APLP2	CDK14	ENHO	GPCPD1	LINGO3	NBEA	PHACTR3	RSU1	SOX6	TSC22D1	
ARHGAP31	CDO1	EPAS1	GPLD1	LMCD1	NCALD	PHLDA1	RTKN	SOX8	TSPAN13	
ARL2BP	CERS4	EPDR1	GPNMB	LNX1	NCEH1	PHLDB1	RUNX1T1	SPON1	TSPAN3	
ARNT2	CHADL	EPN2	GPR37L1	LRP1	NDRG4	PIANP	S100A1	SPRED1	TSPAN6	
ARSB	CHL1	EPS8	GPRASP1	LRRC4B	NELL2	PID1	S100A13	SPRY1	TSPYL4	
ARXES1	CHPT1	ETV4	GPSM2	LRRC4C	NEU4	PLLP	S100A16	SPRY4	TTR	
ARXES2	CHRNA4	ETV5	GPT2	LRRC8D	NFATC2	PLXNB3	SCG5	SSTR1	TXNDC16	
ASCL1	CHST11	FABP7	GRIA3	LRRFIP1	NKX2-2	PNLIP	SCN3A	ST8SIA3	UGDH	

Table S3. Categorized oligodendrocyte associated genes [identified in (29, 30, and 38)].

Committed Oligodendrocyte Progenitor Associated Genes		Newly Formed Oligodendrocyte Associated Genes	Mature Oligodendrocyte Associated Genes
1810041L15RIK	PFN2	CNKSR3	2700046A07RIK
2510003E04RIK	PHYHIPL	GM26834	CDKN1C
2810468N07RIK	PPFIBP1	H2-AB1	DOCK5
3110035E14RIK	RINL	IL23A	KLK6
ABTB2	S100A1	ITPR2	NINJ2
ARSB	S100B	RRAS2	NKX2-9
BCAS1	SEZ6L	SEMA4D	RAB37
BMP4	SIRT2	TMEM163	
BRCA1	SLC1A1	TMEM2	
CD9	SLC22A23		
CDK14	SLC27A3		
CHD3	SLC44A1		
CHN2	SUSD5		
CNP	TIMP4		
CYFIP2	TNR		
CYP2J6	TNS3		
DYNLL1	TRIO		
DYNLL2	USP16		
EDIL3	WASF1		
ENPP6	ZFP365		
EPB41L2			
FYN			
GPR17			
ITPR2			
KAZN			
LIMS2			
MFSD2A			
MPZL1			
MYCL1			
MYRF			
NCAM1			
NEU4			
NKX2-2			
PAK4			
PDCD4			

Table S3. Categorized oligodendrocyte associated genes [identified in (29, 30, and 38)].

Myelin Forming Oligodendrocyte Associated Genes									
1700047M11RIK	B3GALT5	DESI1	FAM214A	IL33	MOBP	PLCL1	SBF1	STK39	UGT8A
2210011C24RIK	BCAR1	DIP2A	FBXO7	INF2	MOG	PLEKHA1	SCARB2	STMN4	UNC5B
2810468N07RIK	BCAS1	DIXDC1	FEZ1	JAM3	MPP5	PLEKHB1	SCCPDH	STXBP3A	VBP1
4931406P16RIK	BIN1	DLG1	FGFR2	JOSD2	MTUS1	PLLP	SCD2	SYPL	VLDLR
5031439G07RIK	BIRC2	DNAJB2	FKBP1A	JPH4	MYO6	PLP1	SEC11C	SYT11	VMP1
AATK	BNIP3L	DNM3	FNBP1	KAT2B	MYRF	PLXNB3	SECISBP2L	SYTL2	WFDC18
ABCA2	BPGM	DOCK1	FRMD8	KAZN	NDRG1	PPP1R14A	SEMA6A	TALDO1	WNK1
ABCA8A	CACNB4	DOCK10	FRYL	KCNA1	NEAT1	PPP1R16B	SEMA6D	TJP2	WSCD1
ACAP2	CAR2	DPY19L1	FTH1	KCNA6	NFASC	PPP2R2A	SEPTIN4	TM7SF3	YBX3
ACOT7	CARHSP1	DPYSL2	GAB1	KCNJ10	NIPA1	PPP2R2C	SEPTIN7	TMBIM1	YPEL2
ADD1	CCDC47	DST	GABARAPL2	KCTD13	NKAIN1	PPP2R3A	SERINC5	TMCC3	YWHAQ
ADD3	CCP110	DUSP26	GAMT	KCTD3	NPC1	PRDX1	SERPINB1A	TMEFF1	ZDHHC20
ADI1	CD82	DUSP3	GATM	KIF1B	NRBP2	PRKACB	SEZ6L2	TMEFF2	ZDHHC9
ADIPOR2	CDC37L1	EDIL3	GDE1	KIF21A	NUDT4	PRKCDBP	SHROOM2	TMEM125	ZFP706
ADO	CDC42BPA	EFCAB14	GIT1	KLF13	OMG	PRKCZ	SIK3	TMEM141	
AGAP1	CDK19	EFHD1	GJB1	KNDC1	OPALIN	PRNP	SLAIN1	TMEM151A	
AGPAT4	CDR2L	EFNB3	GJC2	LAMP1	OSBPL1A	PRR18	SLC12A2	TMEM229A	
ANK	CERS2	EIF4H	GJC3	LAP3	OTUD7B	PRR5L	SLC20A2	TMEM30A	
ANK3	CFL2	ELAVL3	GLOD4	LEPREL4	OXR1	PSAT1	SLC24A2	TMEM63A	
ANKIB1	CHN2	ELOVL1	GLRB	LGI3	PACS2	PTGDS	SLC2A1	TMEM88B	
ANLN	CLDN11	ELOVL7	GLTP	LIMCH1	PAFAH1B1	PTP4A1	SLC38A2	TMEM9B	
ANO4	CLIC4	EMC10	GLUL	LITAF	PAK1	PTPRD	SLC44A1	TMOD2	
APBB1	CLMN	EML1	GNAI1	LPAR1	PCBP4	QDPR	SLC48A1	TNFAIP6	
APLP1	CMTM5	ENDOD1	GNG11	LPGAT1	PCDH9	QK	SLC4A2	TPD52	
APOD	CNP	ENPP2	GOLGA7	MAG	PCNP	RALGDS	SLC6A6	TPPP	
APP	CNTN2	ENPP4	GPM6B	MAL	PDCD6IP	RAP1A	SLC6A9	TPPP3	
ARAP2	CPD	EPB41L3	GPR37	MAP1A	PDE4B	RCBTB1	SLCO3A1	TPST1	
ARC	CPM	EPHB1	GPX4	MAP6D1	PDLIM2	REEP3	SLK	TRF	
ARHGAP23	CPOX	EPS15	GRB14	MAP7	PEA15A	REEP5	SMAD7	TRIM59	
ARID4B	CRYAB	ERBB2IP	GRM3	MAP7D1	PEX5L	RHOG	SMIM15	TSC22D4	
ARPC1A	CSRP1	ERMN	GSN	MAPK8IP1	PHGDH	RHOU	SNAPIN	TSPAN2	
ARRDC3	D1ERTD622E	ETV1	HAPLN2	MAPT	PHLDB1	RNF13	SNX33	TTLL7	
ARSG	DBNDD2	EVI2A	HDAC11	MAST4	PHLPP1	RNF130	SORT1	TTYH2	
ASPA	DDR1	FA2H	HHIP	MBNL2	PHYHIPL	RNF7	SOX2OT	TUBB4A	
ATP1B3	DEB1	FAM134B	HSP90AA1	MBP	PIM3	RTKN	SPOCK3	TULP4	
ATP6AP2	DEGS1	FAM171B	HSPA1A	MCAM	PIP4K2A	RTN4	SPSB1	TYRO3	
ATP8A1	DENND5A	FAM174A	HSPA1B	MID1IP1	PLA2G16	S1PR5	SRCIN1	UBL3	

Supplementary Tables Legends:

Supplemental Table S1: Microglia associated genes [identified in (29-33)] (S1A). Categorized microglia homeostatic genes [identified in (35 and 37)] (S1B). Categorized microglia neurodegenerative genes as identified in studies by [identified in (35-37)] (S1C).

Supplemental Table S2: Categorized astrocyte associated genes [identified in (76)].

Supplemental Table S3: Categorized oligodendrocyte lineage cell associated genes [identified in (29, 30, and 38)].
